# A narrative review on cutaneous manifestations in polycystic ovary syndrome: pathophysiology, diagnosis, management, and psychosocial impact

**DOI:** 10.1097/MS9.0000000000003217

**Published:** 2025-03-28

**Authors:** Muhammad Farhan, Ariana Seyfi, Afra Alnuaimi, Maya Alamour, Sarah Alwarafi, Haya Elastal, Muhammad Hashir Nazir, Balakrishnan Kamaraj, Hrithik Dakssesh Putta Nagarajan, Deborah Delianne, Shyamala Ganesan, Tirath Patel

**Affiliations:** a Ajman University, College of Medicine, Ajman, United Arab Emirates; b Dermatology Department, Sheikh Khalifa Medical Center, Abu Dhabi, United Arab Emirates; c Canadian Specialist Hospital, Dubai, United Arab Emirates; d King Edward Medical University, Lahore, Pakistan; e Madurai Medical College, Tamil Nadu, India; f Department of Internal Medicine, Madurai Medical College, Tamil Nadu, India; g American University of Antigua, Antigua and Barbuda; h Trinity Medical Sciences University School of Medicine, St. Vincent, Saint Vincent and the Grenadines

**Keywords:** polycystic ovary syndrome (PCOS), endocrine disorder, cutaneous manifestation

## Abstract

**Background:**

Polycystic ovary syndrome (PCOS) is a complex endocrine disorder affecting 5%–10% of reproductive-aged women. Its diverse clinical spectrum includes reproductive, metabolic, and dermatological abnormalities, with cutaneous manifestations often serving as visible indicators of underlying hormonal and metabolic imbalances.

**Objective:**

This review explores the pathophysiology, diagnosis, management, and psychosocial impact of the cutaneous manifestations of PCOS, providing an integrated understanding of their clinical significance.

**Methods:**

A comprehensive analysis was conducted based on existing literature to elucidate the underlying mechanisms, diagnostic approaches, and treatment options for dermatological features associated with PCOS, including acne, hirsutism, acanthosis nigricans, seborrheic dermatitis, and androgenic alopecia.

**Results:**

The pathophysiology of cutaneous manifestations in PCOS is driven by hyperandrogenism, insulin resistance, and local androgenic effects on the pilosebaceous unit. Acne and hirsutism are among the most common skin findings, followed by androgenic alopecia and acanthosis nigricans. Diagnostic strategies combine clinical evaluation with hormonal assays and imaging. Management requires a multidisciplinary approach encompassing hormonal therapies, lifestyle modifications, and targeted dermatological treatments. Additionally, these manifestations significantly impair psychosocial well-being, necessitating holistic care.

**Conclusion:**

Cutaneous manifestations are a cosmetic concern and an essential diagnostic and therapeutic focus in PCOS. Addressing these features can enhance patient outcomes by mitigating physical symptoms and improving quality of life. Future research should emphasize personalized treatments and the psychosocial aspects of care to provide comprehensive management for women with PCOS.

**HIGHLIGHTS**

- Cutaneous signs in polycystic ovary syndrome (PCOS) reflect systemic hormonal and metabolic dysfunctions.
- Acne, hirsutism, and alopecia are key dermatological markers in PCOS.
- Management combines hormonal, dermatological, and lifestyle interventions.
- PCOS skin issues significantly impact psychosocial well-being and quality of life.
- Future research should focus on personalized and psychosocial care in PCOS.

## Introduction

Polycystic ovary syndrome (PCOS) is one of the most prevalent endocrine disorders, affecting approximately 5%–10% of reproductive-aged women worldwide^[1]^. The condition was redefined during an expert conference in Rotterdam in 2003, where diagnostic criteria were expanded to include at least two of the following: oligo- or anovulation, clinical and/or biochemical signs of hyperandrogenism, and polycystic ovarian morphology. This broadened definition underscored the diverse clinical spectrum of PCOS, with subsets of patients fulfilling both the classic National Institute of Health criteria and newer phenotypic variations^[2–4]^. PCOS affects a range of at-risk populations, including women with androgenic dermatologic signs like hirsutism, persistent acne, alopecia, menstrual dysfunction; oligo-ovulatory infertility; or insulin resistance^[5]^.

Notably, while a significant proportion of patients with hirsutism have PCOS, the prevalence among those with isolated acne or alopecia is lower^[5]^. The syndrome’s multifaceted presentation encompasses reproductive, metabolic, cardiovascular, psychological, and dermatological sequelae, many of which pose substantial long-term health risks if unrecognized or inadequately managed^[1]^. Cutaneous manifestations, including hirsutism, acne, acanthosis nigricans, and androgenic alopecia, are among the most distressing and clinically significant features of PCOS^[6]^. These signs often serve as visible markers of the underlying endocrine and metabolic abnormalities. Understanding the pathophysiology, diagnosis, and management of these skin conditions is critical, as they frequently influence quality of life (QoL) and provide a gateway for early recognition and intervention in PCOS.

This review integrates current evidence on the cutaneous manifestations of PCOS, exploring their pathophysiology, diagnostic approaches, and management strategies while highlighting the psychosocial implications of this multifaceted disorder. By addressing these dermatological features, the review aims to contribute to a comprehensive understanding of PCOS and its broad clinical impact.

## Pathophysiology of cutaneous manifestations in PCOS

PCOS is a multifaceted endocrine disorder characterized by a complex interplay of hormonal, metabolic, and reproductive abnormalities^[7]^. At its core, the pathophysiology of PCOS involves dysregulation of gonadotropin-releasing hormone (GnRH) secretion, leading to an increased pulsatility of GnRH. This abnormal pattern, often driven by reduced progesterone levels, preferentially stimulates luteinizing hormone (LH) secretion over follicle-stimulating hormone by the anterior pituitary. The resultant imbalance promotes androgen overproduction by ovarian theca cells. These androgens, either directly or via peripheral conversion to dihydrotestosterone (DHT) by 5α-reductase enzymes, play a pivotal role in the development of the cutaneous manifestations seen in PCOS, such as acne, hirsutism, and androgenic alopecia^[8]^.

The pilosebaceous unit (PSU), comprising hair follicles and sebaceous glands, is a primary site affected by hyperandrogenism in PCOS^[9]^. Increased androgen levels stimulate the PSU, enhancing sebum production and promoting hyperkeratinization. Locally elevated DHT levels, resulting from amplified 5α-reductase activity within the PSU, further exacerbate these effects^[9,10]^. This hormonal milieu not only leads to excessive oiliness but also creates an environment conducive to acne vulgaris through follicular plugging and inflammation^[11,12]^. Furthermore, androgen-induced alterations in follicular dynamics shorten the anagen (growth) phase of hair follicles, contributing to androgenic alopecia^[13]^.

Insulin resistance, a hallmark of PCOS, synergistically exacerbates hyperandrogenism^[14]^. Elevated insulin levels amplify androgen synthesis by stimulating ovarian theca cells and suppressing sex hormone-binding globulin (SHBG) production in the liver. This results in an increased availability of free androgens, intensifying their effects on the skin^[15]^. Insulin resistance also indirectly contributes to acanthosis nigricans, characterized by hyperpigmented, velvety plaques commonly observed in PCOS patients. The condition arises from insulin binding to IGF-1 receptors on keratinocytes and fibroblasts, driving epidermal proliferation^[16]^.

Overall, the cutaneous manifestations of PCOS represent the dermatological expression of systemic endocrine dysfunction, highlighting the interconnectedness of hormonal, metabolic, and inflammatory pathways. Understanding these mechanisms is crucial for tailoring effective therapeutic strategies targeting both the systemic and localized effects of PCOS.

## Cutaneous manifestations of PCOS

### Acne vulgaris

Acne vulgaris is a prevalent cutaneous manifestation of PCOS, marked by chronic inflammatory lesions around the pilosebaceous apparatus^[17]^. Unlike pubertal acne, PCOS-related acne predominantly presents with inflammatory papules, pustules, and nodules on the lower face, jawline, chest, and upper back^[18]^. This distribution correlates with areas of increased androgen receptor density^[11]^. The pathogenesis of acne in PCOS is multifactorial but is primarily driven by hyperandrogenism. Elevated androgen levels, such as testosterone and DHT, stimulate sebaceous glands, leading to excessive sebum production^[19]^.

Concurrently, androgen-induced hyperkeratinization contributes to follicular obstruction, forming comedones^[17]^. The overgrowth of propionibacterium acnes, an anaerobic bacterium, triggers local inflammation, further exacerbating acne lesions^[12]^. Resistance to conventional acne therapies, including topical retinoids and antibiotics, may indicate an underlying endocrine disorder like PCOS. Therefore, moderate-to-severe or treatment-resistant acne in women warrants a thorough evaluation for hyperandrogenism and metabolic dysfunctions^[20]^. Early diagnosis facilitates targeted interventions, improving both dermatological and systemic outcomes. While systemic androgen levels may not directly correlate with acne severity, local androgen activity plays a pivotal role^[21]^. Acne prevalence varies across ethnicities, being higher in South Asians and lower in Pacific Islanders^[5]^.

### Hirsutism

Hirsutism, characterized by terminal hair growth in a male-pattern distribution, is one of the hallmark features of PCOS^[5]^. It affects approximately 65%–75% of women with PCOS, with variations influenced by ethnic and individual factors^[4,22]^. This condition results from increased androgen levels acting on the PSU, particularly in androgen-sensitive areas such as the face, chest, and back. Elevated androgen receptor expression and 5α-reductase activity contribute to the excessive hair growth observed in these regions^[9]^.

The severity of hirsutism is commonly evaluated using the modified Ferriman–Gallwey (mFG) score^[22]^. While the degree of hirsutism does not always correlate with androgen excess, its presence warrants a thorough evaluation for underlying hyperandrogenic conditions^[4]^. Diagnostic assessments typically include hormonal assays, ultrasonography for polycystic ovarian morphology^[22]^, and metabolic evaluations as per the AE-PCOS guidelines^[23]^. Effective management requires a multidisciplinary approach, addressing both cosmetic concerns and underlying hormonal imbalances.

### Acanthosis nigricans

Acanthosis nigricans is a characteristic dermatological marker of insulin resistance commonly observed in patients with PCOS. It presents as velvety, hyperpigmented plaques with thickened skin and accentuated lines, primarily in intertriginous areas such as the nape of the neck, axillae, groin, and, inframammary regions^[1]^. Elevated insulin levels in PCOS patients stimulate IGF-1 receptors, driving keratinocyte and fibroblast proliferation, which underpins the development of this condition^[16]^. This manifestation is more prevalent in obese individuals with PCOS and serves as a visible marker of hyperinsulinemia^[24]^. Studies have noted that patients with acanthosis nigricans exhibit significantly higher fasting insulin levels than their counterparts without the condition^[25]^. Its resolution often requires addressing the underlying hyperinsulinemia through weight management and insulin-sensitizing^[1,26]^.

### Androgenic alopecia

Androgenic alopecia, also known as female pattern hair loss (FPHL), is a common cutaneous manifestation of PCOS that reflects the effects of hyperandrogenism on scalp hair follicles. This condition is characterized by a diffuse thinning of hair over the crown while preserving the anterior hairline^[27,28]^. The pathogenesis involves increased DHT activity, driven by 5α-reductase within the dermal papilla, which shortens the anagen phase and miniaturizes hair follicles^[13,29]^. Although androgenic alopecia is associated with PCOS, it does not consistently indicate hyperandrogenism, as other factors such as genetic predisposition, scalp inflammation, and micronutrient deficiencies also contribute^[30]^. A comprehensive evaluation is essential to differentiate androgenic alopecia from other causes of hair loss^[30]^.

### Seborrheic dermatitis

Seborrheic dermatitis (SD) is an inflammatory skin condition often seen in PCOS patients, characterized by erythematous, greasy-looking skin with yellowish scales. It commonly affects seborrheic areas such as the scalp, nasolabial folds, eyebrows, eyelids, and upper chest^[31]^. Androgens play a central role in SD development by increasing sebaceous gland activity^[32]^, which in turn promotes the growth of Malassezia yeast – a key factor implicated in the pathogenesis of the condition. This yeast triggers an inflammatory immune response, further exacerbating SD^[31,33]^. The condition can co-occur with other androgen-driven dermatological issues like acne vulgaris and androgenic alopecia^[33]^. Emerging studies highlight the role of immune cells and genetic pathways like ZNF750/MPZL3 in SD pathogenesis, providing potential avenues for advanced therapeutic interventions^[32]^.

### Skin tags (acrochordons)

Skin tags, or acrochordons, are benign, soft, pedunculated growths that can occur in patients with PCOS, albeit less frequently than other cutaneous manifestations. Studies estimate a prevalence of 9%–10% among PCOS patients^[34,35]^. Their development is linked to insulin resistance, a common hormonal abnormality in PCOS. Elevated insulin levels stimulate epidermal growth, which contributes to their formation. The presence of acrochordons is often an indicator of metabolic dysfunction and warrants a thorough evaluation of glucose metabolism^[34,36]^. While acrochordons are not harmful, they can be cosmetically bothersome.

## Diagnosis of cutaneous manifestations in PCOS

Diagnosing the cutaneous manifestations of PCOS requires a multidisciplinary approach that combines clinical evaluation, laboratory investigations, and imaging studies.

### Clinical assessment

The initial evaluation involves a thorough medical history and physical examination, focusing on specific dermatological symptoms like acne, hirsutism, acanthosis nigricans, and androgenic alopecia. Tools such as the mFG score are employed to quantify hirsutism^[22]^, while dermatoscopic examinations and scalp biopsies may help confirm androgenic alopecia^[30]^. For acne, the distribution, type of lesions, and resistance to conventional treatments may indicate underlying PCOS^[20]^. In cases of acanthosis nigricans, the presence of hyperpigmented plaques, particularly in intertriginous areas, prompts the assessment of insulin resistance^[16]^.

### Laboratory investigations

Hormonal assays are essential for identifying hyperandrogenism. Measuring serum total and free testosterone, along with SHBG, provides insight into androgen bioavailability^[5]^. Advanced methods like liquid chromatography-mass spectrometry are preferred for their precision^[30]^. Evaluating insulin resistance through glucose tolerance tests is crucial, especially in patients presenting with acanthosis nigricans^[1]^.

Additionally, testing for thyroid function, vitamin D, zinc levels, and iron studies may help exclude other conditions contributing to hair loss or skin changes. These tests also support the management of conditions like FPHL, where deficiencies can impair treatment outcomes^[30]^.

### Imaging studies

Ultrasound imaging plays a vital role in confirming the presence of polycystic ovarian morphology, which supports the diagnosis of PCOS. While not specifically linked to cutaneous findings, ultrasound findings provide a critical context for comprehensive patient management^[22]^.

This diagnostic framework ensures an accurate assessment of the diverse skin manifestations of PCOS, enabling effective treatment strategies.

## Management of cutaneous manifestations in PCOS

### General approach to management

Managing cutaneous manifestations in PCOS requires a multifaceted approach addressing systemic and localized factors. A primary focus is on inhibiting androgen production, reducing androgen bioavailability, and countering its peripheral effects. Treatment strategies include:

#### Hormonal therapies

- Combination oral contraceptive pills (OCPs) containing anti-androgenic progestins (e.g. drospirenone, cyproterone acetate) are first-line therapies to reduce androgen levels and regulate menstrual cycles^[1]^.
- GnRH analogs and corticosteroids can also suppress androgen production in selected cases^[1]^.

#### Androgen modulators

Anti-androgens such as spironolactone, flutamide, and finasteride are effective in blocking androgen receptors or inhibiting 5α-reductase. These agents are often combined with OCPs for enhanced efficacy^[1]^.

#### Insulin-sensitizing agents

Metformin and thiazolidinediones are widely used to improve insulin resistance, indirectly reducing hyperandrogenism and associated skin manifestations. These agents are particularly beneficial for patients with acanthosis nigricans or metabolic syndrome^[1]^.

Lifestyle modifications, including dietary changes, weight loss, and physical activity, are foundational elements of management, improving both androgen levels and cardiovascular health^[5]^.

### Specific approaches by manifestation

#### Hirsutism

##### Cosmetic interventions

Hirsutism management often begins with cosmetic procedures aimed at reducing or removing unwanted hair. Traditional methods such as bleaching, plucking, waxing, and electrolysis are frequently employed for mild or localized cases. Laser hair removal is a more advanced option, particularly for extensive or persistent hair growth^[22]^. Alexandrite laser has shown significant efficacy in reducing hirsutism severity and improving psychological outcomes, especially when applied at high fluence. Studies have indicated that the Alexandrite laser is more effective than intense pulsed light (IPL) therapy. Furthermore, combining a diode laser with either metformin or combined OCPs yields superior results compared to a diode laser alone. Similarly, adding metformin to IPL treatment has been found to enhance outcomes^[37]^.

##### Pharmacological therapies

###### Oral contraceptive pills

OCPs are considered the first-line pharmacological treatment for hirsutism, particularly in mild cases^[38]^. OCPs containing ethinyl estradiol combined with anti-androgenic progestins, such as cyproterone acetate, have been found to reduce androgen production and increase SHBG, thereby decreasing free testosterone levels. For example, the use of an OCP containing ethinyl estradiol and drospirenone (DRSP) over 12 cycles resulted in significant reductions in hirsutism scores and free androgen index. This regimen also led to a decrease in plasma testosterone and 17-hydroxyprogesterone, demonstrating its effectiveness in clinical and hormonal control of PCOS-related hirsutism^[39]^

###### Anti-androgens

For moderate to severe hirsutism, anti-androgens such as spironolactone, flutamide, and finasteride are commonly prescribed^[38]^. Spironolactone is frequently used in combination with OCPs, as evidenced by a retrospective analysis involving 200 women with PCOS. The combination of OCPs and spironolactone showed a higher reduction in the mF-G hirsutism score compared to either treatment alone. This approach not only resulted in significant patient satisfaction but also reduced menstrual irregularity and acne^[40]^

###### Metformin

Metformin, an oral antihyperglycemic agent, is another effective option for treating hirsutism in women with PCOS, especially those with insulin resistance. Studies have shown that metformin not only improves insulin sensitivity but also reduces hirsutism severity. A trial by Harborne *et al*^[41]^ demonstrated that metformin was more effective than Dianette (ethinyl estradiol and cyproterone acetate) in reducing hirsutism scores, particularly in patient self-assessment. It also significantly decreased weight, improved cycle regularity, and reduced the growth velocity of hair. These findings highlight the potential role of metformin in addressing hyperinsulinemia, a key factor in PCOS-related hirsutism^[41]^.

###### Bicalutamide

Bicalutamide, an anti-androgen, has also been evaluated for its effectiveness in hirsutism treatment. In a randomized controlled trial (RCT) by Moretti *et al*^[42]^, women with PCOS received either OCPs combined with bicalutamide or OCPs plus placebo for 12 months. The OCP plus bicalutamide group experienced a greater reduction in hirsutism as measured by video-dermoscopy and the mF-G score. No adverse effects were reported except for an increase in total cholesterol and low-density lipoprotein levels. The study concluded that combining OCPs with bicalutamide is well tolerated and more effective than OCP alone in treating severe hirsutism^[42]^.

###### GnRH agonists (GnRH-a)

GnRH agonists such as leuprolide acetate have also been investigated for their efficacy in treating hirsutism. A study comparing leuprolide acetate alone versus leuprolide acetate combined with an OCP containing cyproterone acetate revealed that both treatments significantly reduced hirsutism scores and hair diameter. However, the addition of the OCP resulted in more pronounced androgen suppression, suggesting that combination therapy might be more effective for severe cases^[43]^.

###### Vitamin D supplementation

Vitamin D supplementation has also been explored as a potential treatment for hirsutism in women with PCOS. A study conducted by Al-Bayyari *et al*^[44]^ investigated the effects of vitamin D3 supplementation in overweight women with PCOS and vitamin D deficiency. Over a 12-week period, the treatment group showed significant decreases in total testosterone, parathyroid hormone, free androgen index, and hirsutism scores. These results suggest that vitamin D supplementation may play a supportive role in managing hirsutism in PCOS patients with concurrent vitamin D deficiency^[44]^.

##### Monitoring and outcome assessment

Treatment of hirsutism requires regular monitoring to assess efficacy and make necessary adjustments. The mF-G scoring system is a widely used tool for evaluating hirsutism severity. Periodic reevaluation of this score helps clinicians determine the effectiveness of treatment and the need for modifications. In refractory cases, a combined approach involving cosmetic and pharmacological interventions may be necessary to achieve optimal results^[22]^.

#### Acne vulgaris

##### Overview of acne management

The management of acne in PCOS involves a comprehensive approach targeting sebum production, inflammation, and the proliferation of propionibacterium acnes. For mild to moderate acne, topical agents such as retinoids and antibacterial agents are commonly prescribed to prevent comedone formation. For moderate to severe or refractory cases, oral therapies, including antibiotics, hormonal treatments (OCPs), and isotretinoin, are used. Additionally, light and laser therapies offer adjunctive benefits for inflammatory acne, though they are generally not standalone treatments. Addressing the underlying hyperandrogenism in PCOS is a crucial aspect of long-term acne management. Combined antibiotic and hormonal therapies have shown improved treatment outcomes for resistant *P. acnes*, though the growing threat of antibiotic resistance necessitates cautious and judicious use of antibiotics^[17]^.

##### Pharmacological therapies

###### OCPs and estroprogestins

OCPs are a widely used treatment for acne in PCOS, particularly for cases driven by androgen excess. Colonna *et al*^[45]^ conducted a study to evaluate the impact of two estroprogestins, ethinyl-estradiol (EE) combined with drospirenone (DRSP) and EE paired with chlormadinone acetate (CMA), on serum androgen concentrations and skin characteristics. The study, which included 59 women with mild to severe acne, demonstrated that both treatments were effective and well-tolerated, leading to significant improvements in skin and hormonal parameters. The EE/DRSP combination showed a more pronounced reduction in acne compared to EE/CMA, suggesting it may be a more potent therapeutic option for PCOS-related acne^[45]^.

###### Anti-androgens (spironolactone)

Spironolactone, a well-known anti-androgen, is effective in treating acne by reducing sebum production. Its use is especially beneficial in PCOS patients with hyperandrogenism. However, careful monitoring is required due to potential systemic effects, such as alterations in electrolyte balance and menstrual irregularities. Spironolactone’s role in acne management is often complementary to other treatments, such as OCPs or topical agents^[46]^.

###### Metformin

Metformin has shown significant benefits in treating acne in women with PCOS, especially in cases associated with insulin resistance. A meta-analysis by Yen *et al*^[47]^ included 51 studies on 2405 PCOS patients and revealed that metformin as an adjunctive therapy significantly improved acne scores compared to treatments without metformin. The study also found that the presence of acne significantly decreased following metformin treatment. Moreover, pooling pretreatment and posttreatment data indicated a significant reduction in acne scores after metformin use (standardized mean deviation [SMD]: −0.712; 95% CI −0.949 to −0.476), supporting its role in managing PCOS-related acne^[47]^.

###### Acarbose

Acarbose, an alpha-glucosidase inhibitor, has also been investigated for its role in managing acne in hyperinsulinemic women with PCOS. In an RCT by Ciotta *et al*^[48]^, women with hyperinsulinemia and PCOS were randomly assigned to receive either acarbose or a placebo for three months. The results showed a significant reduction in the acne score in the acarbose group, while no significant changes were observed in the placebo group. These clinical improvements were accompanied by a significant reduction in the insulin response to a glucose load, decreased serum LH, total testosterone, androstenedione, as well as an increase in SHBG concentrations. This evidence suggests that acarbose can be an effective option for reducing acne in PCOS patients with hyperinsulinemia^[48]^.

##### Adjunctive and supportive therapies

Light and laser therapies have been explored as adjunctive treatments for acne. These therapies target inflammation and reduce *P. acnes* colonization, offering additional benefits in the management of inflammatory acne. However, they are not typically used as standalone treatments and are often combined with pharmacological approaches for optimal outcomes^[17]^.

##### Endocrinological considerations

Addressing the underlying hormonal imbalances in PCOS is essential for effective acne management. Therapies aimed at reducing androgen production or increasing SHBG can help decrease sebum production and acne severity. Endocrinological therapies, including the use of anti-androgens, OCPs, and insulin-sensitizing agents like metformin and acarbose, offer long-term benefits in managing PCOS-related acne. The combination of antibiotics and hormonal therapies can enhance treatment efficacy in resistant cases of *P. acnes*, but the growing concern about antibiotic resistance highlights the need for prudent use of antibiotics in clinical practice^[20]^.

#### Acanthosis nigricans

The management of acanthosis nigricans focuses on correcting hyperinsulinemia and addressing the underlying insulin resistance. Lifestyle modifications, including weight reduction through dietary changes and increased physical activity, form the cornerstone of therapy. Pharmacological interventions, such as insulin-sensitizing agents like metformin and thiazolidinediones, have proven effective in improving insulin sensitivity and reducing lesion severity^[1,26]^. Topical treatments, including keratolytics like ammonium lactate and retinoids, provide symptomatic relief by softening and thinning hyperkeratotic plaques^[1,49]^. Calcipotriol, a vitamin D3 analog, has shown promise in reducing epidermal proliferation associated with acanthosis nigricans^[49]^. However, sustained improvement often requires long-term metabolic control, as hyperinsulinemia drives lesion recurrence^[1]^.

#### Seborrheic dermatitis

SD in PCOS is managed through topical and systemic treatments aimed at reducing inflammation and controlling *Malassezia* yeast overgrowth. Topical antifungal agents, such as ketoconazole, ciclopirox olamine, and selenium sulfide, are the mainstays of therapy. For more severe cases, systemic antifungal agents or anti-inflammatory therapies may be required^[33]^. Adjunctive measures include maintaining skin barrier integrity and addressing underlying androgenic stimulation of sebaceous glands.

#### Skin tags (acrochordons)

Skin tags, though less common in PCOS, are managed primarily through procedural interventions. Techniques such as cryotherapy^[50]^, excision using tissue forceps^[51]^, or topical application of agents like oregano essential oil^[52]^ have shown efficacy in removing these lesions. Studies indicate that cryotherapy offers high patient satisfaction with significant lesion clearance^[50]^.

The development of acrochordons is closely linked to insulin resistance^[34]^; thus, addressing hyperinsulinemia with agents like metformin or lifestyle modifications can prevent recurrence. Comprehensive management should include screening for metabolic abnormalities to mitigate associated health risks.

#### Androgenic alopecia

The treatment of androgenic alopecia in PCOS involves both systemic and topical therapies. Anti-androgens, such as spironolactone and finasteride, are commonly used to block androgen effects on hair follicles. These agents may be combined with oral contraceptives to enhance therapeutic outcomes. Topical minoxidil (5%) is a cornerstone of therapy, prolonging the anagen phase of hair growth and improving hair density. It is often used alongside anti-androgens for better efficacy^[30,53]^.

Emerging therapies like platelet-rich plasma (PRP) injections and low-level laser therapy have shown modest benefits. PRP promotes follicular regeneration through growth factors, while laser therapy enhances scalp circulation. Hair transplantation can be considered for advanced cases, but it should be accompanied by ongoing medical therapy to maintain results^[30]^.

Although not directly related to management, addressing the psychosocial impact of hair loss is critical. FPHL in PCOS is often associated with emotional distress, necessitating a holistic approach that includes psychological support alongside medical treatment.

## Psychosocial impact and QoL

The cutaneous manifestations of PCOS significantly impact the psychosocial well-being and QoL of affected individuals. Hirsutism^[54]^, acne^[55]^, and androgenic alopecia^[56]^, often lead to considerable emotional distress, low self-esteem, and social withdrawal. Studies highlight that hirsutism has a pronounced negative effect on QoL, with levels of anxiety and depression correlating with the severity of the condition. Although not life-threatening, these symptoms contribute to profound social and emotional disability, underscoring the importance of effective medical treatment paired with psychological support^[54]^.

Androgenic alopecia, in particular, is linked to moderate impairments in health-related QoL (HRQoL). Younger individuals and those with more severe hair loss report greater emotional and social challenges, including difficulty maintaining personal relationships and professional opportunities. The significance of hair in self-identity amplifies the psychological burden, especially among employed and educated individuals facing societal pressures^[56,57]^.

Acne vulgaris is another condition with a marked psychological impact, often associated with increased anxiety, depression, and social isolation, regardless of acne severity. The condition’s prevalence among adolescents and young adults makes them especially vulnerable to the emotional repercussions^[58,59]^.

PCOS itself is associated with a notable reduction in HRQoL, affecting both physical and mental health domains. Women with PCOS frequently report lower life satisfaction, with symptoms and excess weight being major contributors^[60,61]^. Adolescents with PCOS face additional challenges, with their QoL significantly reduced compared to healthy peers. Mental distress in PCOS patients is comparable to that observed in chronic conditions such as asthma and depression, necessitating a multidisciplinary approach to care^[61,62]^.

## Future directions

The growing understanding of the interplay between hormonal, metabolic, and dermatological factors in PCOS offers promising avenues for future research and management^[63]^. Advances in molecular biology may enable the development of targeted therapies addressing specific pathways, such as androgen receptor modulation, 5α-reductase inhibition^[46]^, and immune regulation in conditions like SD^[32]^. Emerging treatments like PRP therapy for androgenic alopecia^[30]^ and innovative topical or systemic agents for acne^[17]^ and acanthosis nigricans^[1]^ highlight the potential for more personalized and effective interventions.

Beyond clinical treatment, the psychosocial impact of PCOS underscores the importance of integrating psychological support into management plans. Holistic care models addressing both physical symptoms and emotional well-being could significantly improve QoL^[54]^. Additionally, ongoing research into the metabolic and cardiovascular risks associated with PCOS emphasizes the critical need for early diagnosis and intervention^[64]^. Future prospects lie in combining endocrinological, dermatological, and psychological expertise to provide comprehensive care tailored to individual patient needs.

## Conclusion

The cutaneous manifestations of PCOS are key indicators of the underlying hormonal and metabolic dysfunctions associated with this complex endocrine disorder. Conditions such as acne, hirsutism, androgenic alopecia, and acanthosis nigricans not only impact physical appearance but also significantly affect psychosocial well-being and QoL. Understanding the pathophysiological mechanisms behind these manifestations is crucial for accurate diagnosis and effective management. A multidisciplinary approach that integrates hormonal therapies, lifestyle interventions, and targeted dermatological treatments is essential for addressing both the systemic and cutaneous aspects of PCOS. By prioritizing early recognition and comprehensive care, clinicians can improve outcomes and reduce the long-term health burden faced by women with PCOS. Future research should focus on personalized therapies and addressing the psychosocial dimensions to ensure holistic management of this multifaceted condition.

## Data Availability

Data is publicly available.
